# *Aspergillus fumigatus*, One Uninucleate Species with Disparate Offspring

**DOI:** 10.3390/jof7010030

**Published:** 2021-01-06

**Authors:** François Danion, Norman van Rhijn, Alexandre C. Dufour, Rachel Legendre, Odile Sismeiro, Hugo Varet, Jean-Christophe Olivo-Marin, Isabelle Mouyna, Georgios Chamilos, Michael Bromley, Anne Beauvais, Jean-Paul Latgé

**Affiliations:** 1 Unité des Aspergillus, Institut Pasteur, 75015 Paris, France; francois.danion@chru-strasbourg.fr (F.D.); isabelle.mouyna@pasteur.fr (I.M.); beauvais.latge@gmail.com (A.B.); 2 Centre d’infectiologie Necker Pasteur, Hôpital Necker-Enfants Malades, 75015 Paris, France; 3 Department of Infectious Diseases, CHU Strasbourg, 67000 Strasbourg, France; 4 Manchester Fungal Infection Group, University of Manchester, Manchester M13 9PL, UK; norman.vanrhijn@manchester.ac.uk (N.v.R.); mike.bromley@manchester.ac.uk (M.B.); 5 Bioimage Analysis Unit, Institut Pasteur, CNRS UMR3691, 75015 Paris, France; alexandre.dufour@gmx.fr (A.C.D.); jean-christophe.olivo-marin@pasteur.fr (J.-C.O.-M.); 6 Centre de Ressources et Recherches Technologiques (C2RT), Institut Pasteur, Plate-Forme Transcriptome et Epigenome, Biomics, 75015 Paris, France; rachel.legendre@pasteur.fr (R.L.); odile.sismeiro@pasteur.fr (O.S.); hugo.varet@pasteur.fr (H.V.); 7 Département Biologie Computationnelle, Hub de Bioinformatique et Biostatistique, Institut Pasteur, USR 3756 CNRS, 75015 Paris, France; 8 Institute of Molecular Biology and Biotechnology FORTH and School of Medicine, University of Crete, 70013 Heraklion, Crete, Greece; hamilos@uoc.gr

**Keywords:** *Aspergillus fumigatus*, conidium, germination, asynchrony, transcriptome, aspergillosis

## Abstract

Establishment of a fungal infection due to *Aspergillus fumigatus* relies on the efficient germination of the airborne conidia once they penetrate the respiratory tract. However, the features of conidial germination have been poorly explored and understood in this fungal species as well as in other species of filamentous fungi. We show here that the germination of *A. fumigatus* is asynchronous. If the nutritional environment and extensive gene deletions can modify the germination parameters for *A. fumigatus*, the asynchrony is maintained in all germinative conditions tested. Even though the causes for this asynchrony of conidial germination remain unknown, asynchrony is essential for the completion of the biological cycle of this filamentous fungus.

## 1. Introduction

*Aspergillus fumigatus* is a saprotrophic fungus which lives in the soil in decaying vegetal material and upon inhalation of airborne conidia can cause life-threatening infections. Conidia are ubiquitous in the air and continuously transported through the air current. Encountering an aqueous nutritive environment leads to conidial germination. Morphologically, germination can be classically divided into three stages. First, a lag phase occurs without any morphological modification but with an active intracellular expression of genes [1,2], preparing the synthesis of protein necessary for germination but not requiring de novo RNA synthesis. The second stage is the isodiametral growth of germinating conidia consecutive to an intracellular increase in osmotic pressure. The third stage corresponds to a polarized growth with the formation of a germ tube [3,4] after the first mitosis. If the association between the polarized growth and mitotic events has been well established in *Aspergillus* [4,5,6], the characterization of the early stages of germination has been poorly understood. Even though an *Aspergillus* colony originates from one conidium with a single nucleus, heterogeneity in the physiological activities in different regions of a colony or in different hyphal compartments within a single hyphae has been repeatedly noticed [7]. This heterogeneity is also observed for a conidial population and linked to resistance to antifungal drugs [8]. Bleichroldt et al. [9] identified distinct subpopulations of *A. fumigatus* conidia showing different patterns of heterogeneity at the cell wall level that manifest differential fitness to the antifungal caspofungin. However, the exact mechanisms linking single cell heterogeneity to this drug susceptibility status are not understood.

Although germination is the primary event at the origin of human infections, exit of quiescence and germination of the conidia have been poorly documented. This is especially true for the pre-mitotic events leading to the swelling of the conidium. Moreover, even though germination is highly dependent on the nutritional environment [10], the role of different nutrients during early germination stages has not been precisely investigated yet. The aim of our study was to characterize the heterogeneity and asynchrony of the early steps of the germination process of the filamentous fungi *A. fumigatus* in different nutritional environments.

## 2. Methods

### 2.1. Strains

The *A. fumigatus* strain used in this study was CEA17ΔakuBKU80, which originated from the clinical isolate CBS 144–89 [11,12]. This strain was maintained on 2% malt-agar slants at ambient temperature. Preliminary experiments showed that the percentage of germination and the size of the swollen conidia when the germ tube was issued were not significantly different with 2 to 4 weeks old conidia (data not shown). Accordingly, conidia were harvested from 21-day-old malt-agar slants using 0.05% Tween–water, filtered (40 µm), washed three times with 0.05% Tween–water and then counted using the LUNA Automated Cell Counter.

Several cell wall mutants of the Aspergillus unit of the Institut Pasteur and a library of transcription factor mutants previously constructed and provided by the Bromley laboratory were also used in this study. The cell wall mutants were affected in their content of melanin, α1,3 glucan, rodlets, galactomannan and chitin [13,14,15,16,17].

### 2.2. Germination Conditions

Conidia were suspended in different media at a concentration of 5 × 10^5^ spores/mL in a volume of 220 µL in 8-well uncoated Ibidi slides (80821) and incubated at 37 °C. Four media were used: (1) D-glucose 1% (G); (2) D-glucose 1% with ammonium tartrate dibasic 0.92 g/L (GA); (3) D-glucose 10g/L with ammonium tartrate dibasic 0.92 g/L and KH_2_PO_4_ 1.52 g/L (GAP); (4) Minimal medium (MM). Minimal medium contains D-glucose 10 g/L, ammonium tartrate dibasic 0.92 g/L, KH_2_PO_4_ 1.52 g/L, MgSO_4_ 0.52 g/L, KCL 0.52 g/L, trace element solution (Na_2_B_4_O_7_, CuSO_4_, MnSO_4_, Na_2_MoO_4_, ZnSO_4_). All 4 media were supplemented with 34.5 g/L MOPS (morpholinepropanesulfonic acid; pH 7) to eliminate the possible change in pH during germination. Ammonium, glucose and phosphate are the three essential macronutrients for the synthesis of all fungal macromolecules and hence required for *Aspergillus* growth. However, their separate or joint impact has not been assessed during the germination of *A. fumigatus*. This is the reason for the selection of the G, GA and GAP media in comparison with the complete defined medium MM. In addition, RPMI-1640 medium is classically used in the testing of the effect of antifungal drugs and selected for its composition, which is closer to the host environment, so it has been also used [18,19,20].

### 2.3. Video Microscopy

The germination process was followed over a 24-h time period under a Nikon TI microscope equipped with a 37 °C incubator and a 40× objective, with 1 picture captured every 4 min. Data were analyzed using ICY software [21,22,23]. We set up a specific program to automatically detect the conidia and measure their size over time. The size was evaluated as the area measured in pixels and was kept as such since pixels were the true measures recorded by the software. For a better understanding of the size of the fungal cell, pixels were sometimes translated into µm^2^ with a value of 0.0256 µm^2^ per pixel.

### 2.4. Nucleus and Septum Staining

Staining for nucleus and septum was performed on unfixed material. For nuclear staining, supernatant of the germinating medium was removed and Hoechst (Molecular probes/Thermofischer 33342) was added at a concentration of 1:2.000 in 220 µL in phosphate-buffered saline (PBS) for 10–15 min, then washed and resuspended in PBS. The number of nuclei was counted with an Evos microscope using bright field and UV filters.

For septum staining, CalcoFluor White (CFW) was added at a concentration of 5 µg/mL for 10–15 min, washed and then evaluated with an Evos microscope using bright field and UV filters.

Incubation time was 24 h in G, GA and GAP media and 11 h in MM medium to obtain the same morphology with short germ tubes.

### 2.5. Transcriptome

The amount of DNA extracted from germinating conidia was very low in G and GA media, with a mean of 2 ng in GA and 28 ng in G for 108 conidia germinating at a 40 ± 10% ratio, and did not permit us to run a proper RNA-seq experiment. Accordingly, the transcriptome analysis was only performed in GAP medium and MM medium. Even though the composition of the culture media remained the same, the experimental conditions were slightly different from the conditions used for the video microscopy to be able to recover enough mRNA to perform the RNA-seq experiment. For the transcriptome experiments, the fungus was grown in square plastic dishes of 120/120 mm (Greiner, Kremsmünster, Austria) with 35 mL of liquid medium, with a time of incubation of the conidia in the GAP medium and MM medium of, respectively, 30 h of incubation in GAP medium and 11 h for MM medium to maintain a similar fungal morphology in both conditions. Moreover, the number of germinated conidia presenting a germ tube was 40 ± 10% in both media. However, in MM medium, the fungus continued to grow after 11 h, whereas in GAP medium, growth stopped after 30 h. Three biological replicates were performed. Conidia were collected in phenol and then disrupted with 0.5-mm diameter glass beads in a volume of 500 µL. RNA was isolated using the Ambion TURBO kit. Total RNA was checked on the Bioanalyser system (Agilent) for its quality and integrity. Ribosomal RNA depletion was performed using the Bacteria RiboZero kit (Illumina, San Diego, CA, USA). From rRNA-depleted RNA, directional libraries were prepared using the TruSeq Stranded mRNA Sample Preparation Kit, following the manufacturer’s instructions (Illumina, San Diego, CA, USA). Libraries were checked for quality on Bioanalyser DNA chips (Agilent, Santa Clara, CA, USA). Quantification was performed with the fluorescent-based quantitation Qubit dsDNA HS Assay Kit (Thermo Fisher Scientific, Waltham, MA, USA). Sequencing was performed as an SRM run (SR: single read, M: multiplexed samples) for 65-bp sequences on a HiSeq 2500 Illumina sequencer (65 cycles). The multiplexing level was 6 samples per lane. Quality control of FastQ files was performed using FastQC and trimmed with Trimmomatic (sliding window, averaging over 4 bases with average quality of >20) [24]. Trimmed reads were aligned to the *A. fumigatus* A1163 genome (assembly ASM15014v1, Ensembl Fungi release 48) via HISAT2 (standard parameters) and counted with FeatureCounts with the Ensembl Fungi v48 gene annotation [25,26,27]. Differential expression analysis was performed with DESeq2 [28]. A generalized linear model was set in order to test for the differential expression between the intracellular persisters and control conditions. Raw P values were adjusted for multiple testing according to the Benjamini and Hochberg procedure and genes with an adjusted p value lower than 0.05 were considered differentially expressed. RNA-seq datasets are available in the NCBI Gene Expression Omnibus under accession number GSE152682 (https://www.ncbi.nlm.nih.gov/geo/query/acc.cgi?acc=GSE152682).

### 2.6. Epigenetic Inhibitors

Epigenetic inhibitors were selected from previous publications, some of them having already been shown to be active against *A. fumigatus* [29,30,31,32,33,34,35,36,37,38]. All inhibitors were added to G, GA, GAP and MM media with successive dilutions from the starting solution. Epigenetic inhibitors were RG108 (diluted in DMSO at 70 mg/mL), Vorinostat diluted in DMSO at 2 mg/mL, Bromidespin in DMSO 10 mg/mL, Zebularine in DMSO at 50 mg/mL, Trichostatin in DMSO at 5 mM, Nanaomycin A in DMSO at 13 mg/mL, JQ1 in DMSO at 5 mg/mL, Terbinafin in DMSO at 10 mg/mL 3,-deazaneplanocin in water at 3 mg/mL, GSK1324726A: in DMSO at 50 mg/mL. Conidial germination as well as its asynchrony was assessed under a light microscope.

### 2.7. Statistical Analysis

Data were analyzed using one or two-way ANOVA on 20–100 conidia measured per factor. Least mean square classification was used to rank average data with a Student *t* test and means not connected by the same letter are significantly different. Analysis was run on JMP software (SAS Institute, Cary, NC, USA).

## 3. Results

### 3.1. Characterization of Conidial Germination in A. fumigatus

**Figure 1:**
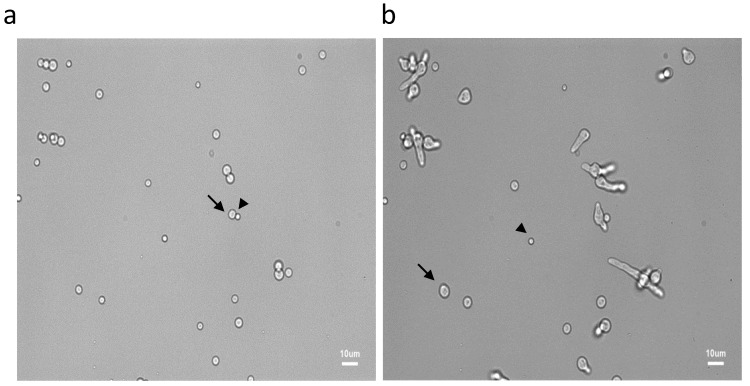
Asynchrony of conidial germination after 6 h (**a**) and 9.30 h (**b**) of incubation in the MM medium. See the resting (arrow heads), swollen (arrows) and germinated conidia

**Figure 2:**
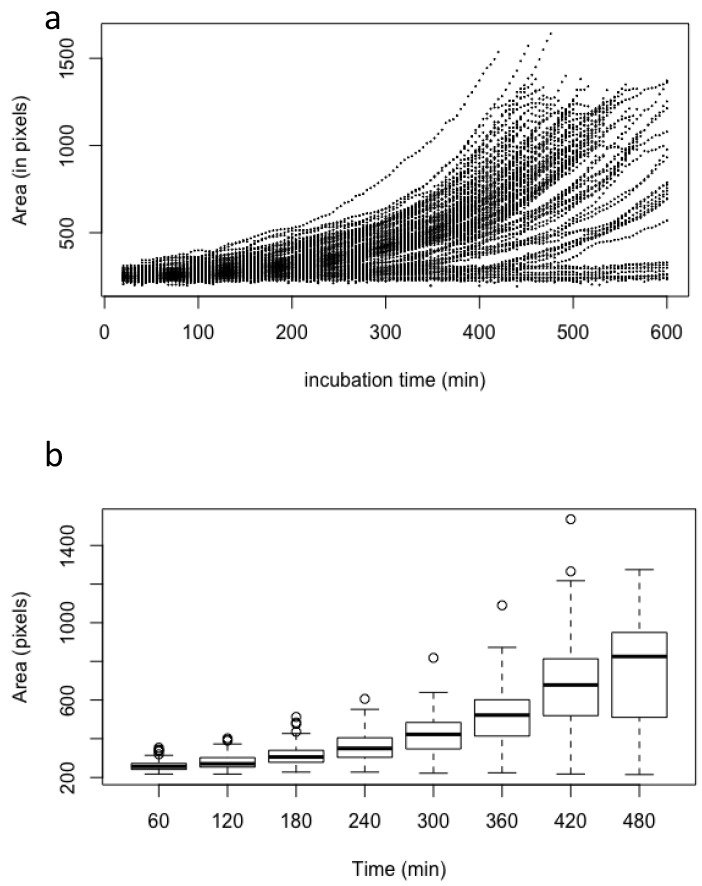
Asynchrony of conidial germination estimated by the measurement of the conidial volume of 100 conidia after incubation in the MM medium; (**a**) scatter plot of the volume of each conidia measured every 4 min; (**b**) box plot of the volume of all conidia measured every 60 min. Volume (estimated as pixels) was measured only on conidia without germ tubes.

Germination of fungal conidia is initiated by an isodiametral growth stage resulting in the formation of a spherically enlarged, “swollen” conidium. This is followed by a polarized phase leading to the production of a germ tube. As previously reviewed by other authors and ourselves [1,4], and from data presented herein, it is clear that germination of *A. fumigatus* conidia is asynchronous. In MM medium, swelling of the first conidia started after around 1 h, whereas others did not reach this stage until 10 h post-incubation (Figure 1 and Figure 2). During the swelling phase, conidial size increased from a mean area of 254 ± 17 pixels (6.5 µm^2^) for resting conidia to an area of 757 ± 283 pixels (19.4 µm^2^) after 8 h. Average size and SD were computed on 100 conidia to analyze the asynchrony of the conidial germination. Among the conidial population, the first germ tube was formed 7 h after the beginning of the incubation in MM medium, whereas the last one appeared after 18 h. After 11 h, more than 90% of conidia had germ tubes. The number of germ tubes per conidium also varied among conidia: 16 h after the beginning of germination, 4% of conidia had one germ tube, 51% had two germ tubes, 44% had three germ tubes and 2% had four germ tubes.

The kinetic of growth from the beginning of the swelling to the emergence of the first germ tube was exponential (i.e., proportional to 𝑒^𝑟^), with an intrinsic growth rate 𝑟 of 0.0035 ± 0.00049 (standard deviation) every 4 min. In our experimental conditions, the lag phase had a mean of 167 min (±85 for standard deviation). The value of the intrinsic growth rate is an inherent property of each conidium which is not influenced by the length of the lag phase or the time at which the germ tube emerged (Figure 2; Supplementary Figure S1), indicating that the germination characteristics are an intrinsic property of each conidium.

### 3.2. Does the Nutritional Environment Modify the Conidial Germination Asynchrony?

**Figure 3:**
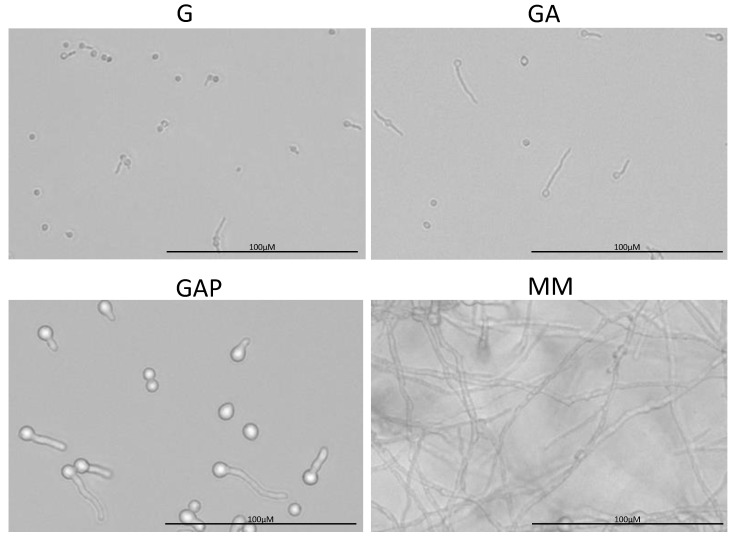
Morphology of the conidia germinating in G (glucose), GA (glucose + ammonium tartrate), GAP (glucose + ammonium tartrate + phosphate) and complex MM for 24 h.

To assess if asynchrony of germination is linked to specific nutrients in the growth media, germination of the conidia was investigated in four different culture conditions: glucose alone (G), glucose and ammonium (GA), glucose, ammonium and phosphate (GAP) and minimal medium (MM) (Table 1; Figure 3; Supplementary Figures S2–S6). Even though the value of the different germination parameters was directly dependent on the environmental culture conditions (Table 1), the asynchrony of the conidial germination was conserved in the G, GA, GAP, MM (Figure 3) and RPMI media.

**Figure 4:**
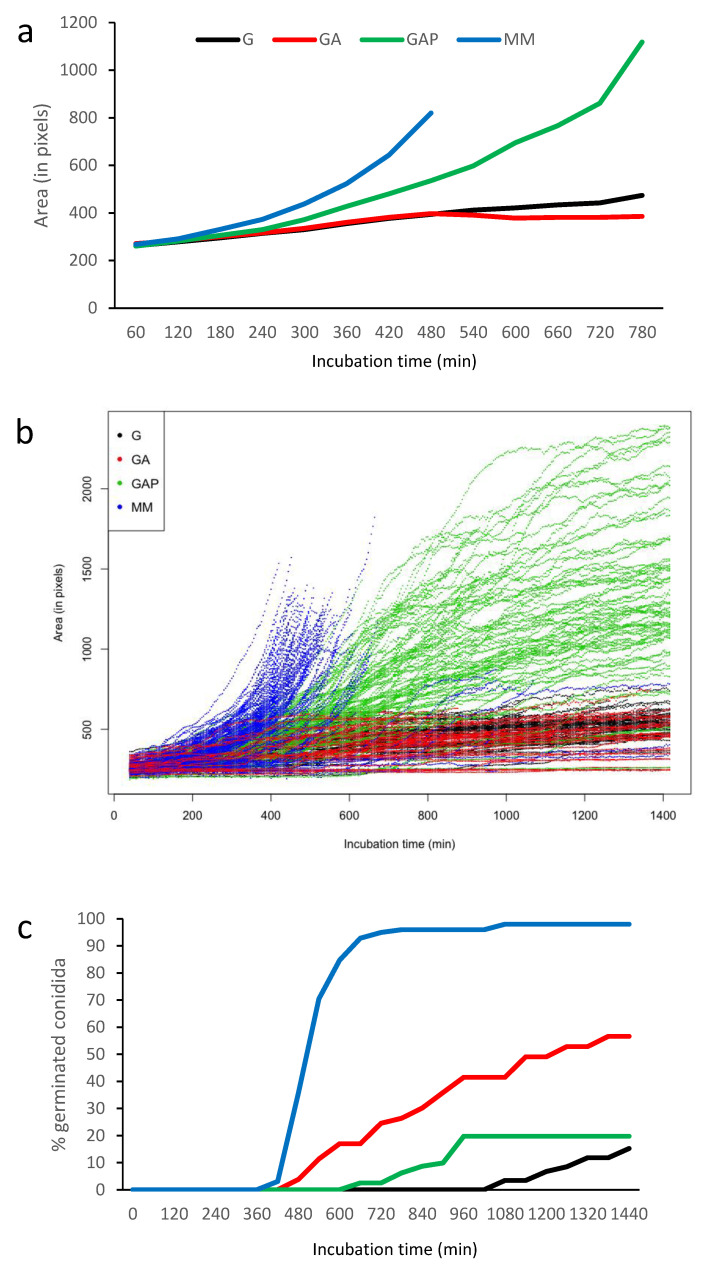
Quantification of the conidium germination in G, GA, GAP and MM media. (**a**) Average size in the different media until the emission of the first germ tube (computed as conidial surface estimated by pixels); (**b**) heterogeneity of germination with 100 swollen conidia (until the emission of the first germ tubes) randomly selected; (**c**) percentage of germinated conidia with germ tubes.

Conidial swelling was not correlated with the percentage of germinating conidia. The largest increase in the size of the swelling conidia was seen in MM medium but also in GAP medium, while the percentage of germination was the lowest in GAP medium (Figure 4; Supplementary Figure S6; Table 1). In G medium, the size of conidia at 8 h was small, with 395 ± 10 px (10.1 µm^2^). At 16 h, mean size of swollen conidia was 471 ± 10 px (12.1 µm^2^). In GA, conidia were also small, with a mean size of 412 ± 13 px (10.5 µm^2^) at 8 h of incubation and 460 ± 16 px (11.8 µm^2^) at 16 h in swollen conidia. In GAP medium, the phenotype was completely different, with conidial swelling similar to that seen in MM medium. At 8 and 16 h, mean sizes of conidia (without germ tubes) in GAP were 518 ±13 px (13.3 µm^2^) and 1078 ± 24 px (27.6 µm^2^), respectively. The size of the conidia after 6 h incubation in RPMI was 704 ± 27 px and the first germ tube appeared the earliest of all media (375 ± 7 min), with a mean size of 906 ± 25 px.

The first germ tube was seen after 7 h of incubation in MM, 8 h in GA, 11 h in GAP and 18 h in G (Figure 4). With the exception of GAP, the number of germinated conidia slowly increased over time to reach more than 80% after 10 h in MM and 2 days in GA and G. In GAP, the emergence of new germ tubes unexpectedly stopped after 24 h and never reached a percentage above 20% of germinated conidia in the experimental conditions used for video microscopy.

**Figure 5:**
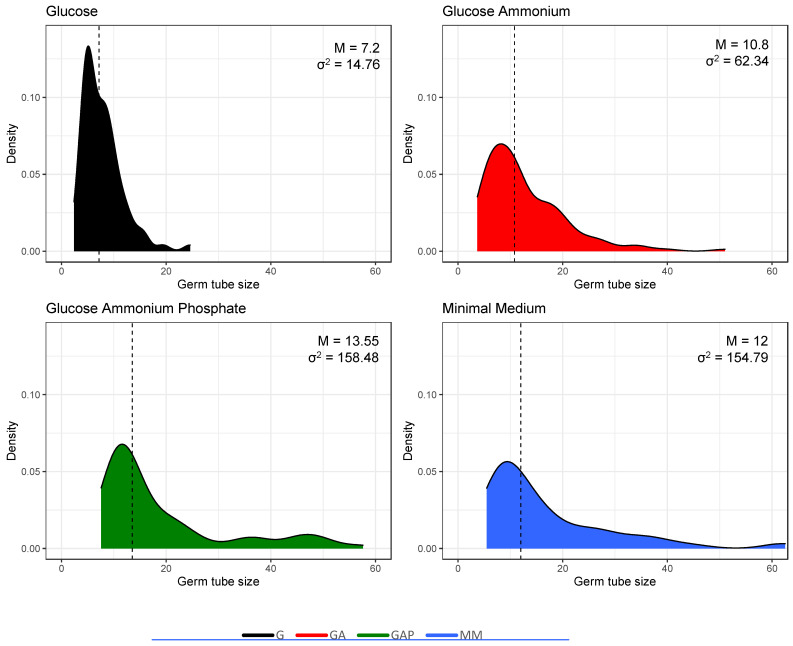
Heterogeneity in the population of germinated conidia estimated by the size of the germ tubes (in µm) in the different media. Incubation time was 24 h in G, GA, GAP and 11 h in MM. Median values and variance are shown in the upper right corner of each plot.

The morphology of the germ tube was also different in the different media (Figure 3). Germ tubes were thin in G and GA media, with a diameter of 0.9 µm, compared to 2.6 µm in the GAP and MM media. In G medium, germ tubes were short, with an average length of 7.5 µm and 12 µm after, respectively, 24 and 48 h. In GA, germ tubes were longer than in G medium (average length of 17 and 29 µm after 24 and 48 h). In GAP, germ tubes had an average length of 14 and 15 µm after, respectively, 24 and 48 h due to growth arrest after 30 h. The largest germ tubes with a diameter of 2.6 µm were similar in MM and GAP media (Table 1). Interestingly, the analysis of the germ tube size also shows heterogeneity of the population of the germinated conidia (Figure 5). Additional experiments in a medium with glucose and phosphate without ammonium tartrate indicated that increased width of the germ tube was associated with the presence of phosphate (data not shown).

After 24 h of incubation in the medium, the number of germ tubes per conidium was influenced by the composition of the medium. Only single germ tubes were seen in G. In GA and GAP media, 3.9% and 13.7% of germinated conidia displayed two germ tubes after 24 h. In MM medium, 25% of the germinated conidia had two germ tubes after 11 h of incubation in the medium (data not shown).

### 3.3. Nuclear Division and Septum Formation in Different Nutritive Environments

**Figure 6:**
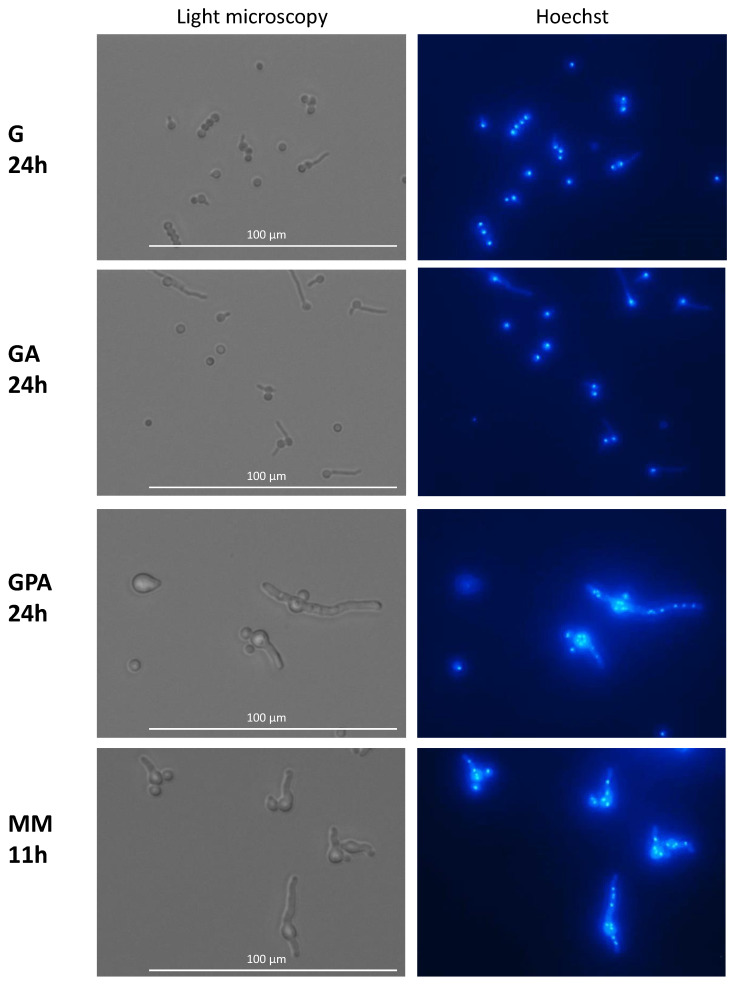
Nuclei in germinating conidia. Germinated conidia in G and GA media only displayed one nucleus per conidium after 24 h of germination. Active nuclear division in MM and GAP media after respectively 11 h and 24 h germination. Staining with Hoechst of germinating conidia; Nucleus staining by Hoechst.

In G and GA media after 24 h of germination, all conidia, even when they had a germ tube, had a single nucleus which eventually migrated into the germ tube (Figure 6). After 3 days of incubation in G and GA (at a time when growth had stopped), only 4% of the germinated conidia had two and 2% had three nuclei, indicating that mitosis was inhibited. This limited increase in the number of nuclei was independent of the length of the germ tube (data not shown). At this time, the nucleus stayed in >80% of the germinated conidia in the original conidium and did not migrate into the germ tube, indicating that the nuclear migration was inhibited concomitantly with the repression of mitosis. In the remaining germinated conidia, one nucleus was seen in the germ tubes. In contrast to G and GA media, mitosis was active in GAP and MM media; the number of nuclei started to increase during the conidial swelling and continued to expand during germ tube elongation. Interestingly, the number of nuclei per conidium before germ tube initiation or in germinated conidia in GAP (mean of 3 nuclei) was higher than in MM (mean of 2 nuclei). Later during germ tube emergence, an average of 7 to 8 nuclei was observed in 30 to 35 µm germ tubes in MM (after 11 h incubation), whereas 10 nuclei were present in germ tubes of similar size in GAP (after 24 h incubation) (data not shown).

**Figure 7:**
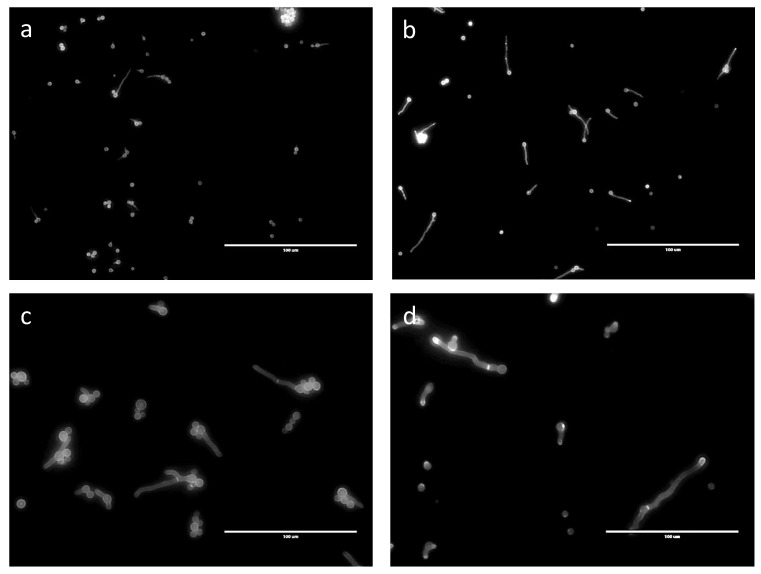
Septa shown by CalcoFluor staining of germinating conidia in different media. (**a**) G; (**b**) GA; (**c**) GAP; (**d**) MM.

No septa were seen in germ tubes produced in G medium after 24 h (Figure 7). Even though germinated conidia had only one nucleus in GA after 24 h, 6.6% of the germ tubes, which ranged in size from 15 to 35 µm, had a septum. In GAP (24 h) and MM (11 h), 11% and 12% of the germ tubes, respectively, had septa, even though the average length of the germ tubes (12 to 60 µm in both media) was similar to those in the GA medium (Figure 7) and even though the numbers of nuclei were much higher in the GAP and MM media (see above).

### 3.4. Growth Arrest in G, GA and GAP Media Is Caused by Exhaustion of Nutrients Present in the Conidia

In our experimental conditions, germ tube growth stopped after 24–30 h in GAP media and 48 h in G media and GA media, while the germ tube continued to grow in MM medium. After the arrest of growth, the replacement of G, GA and GAP medium by the same respective fresh medium did not induce renewed growth. However, when G, GA and GAP media were replaced by MM medium, growth was immediately restored (Supplementary Figure S7). These results indicate that the growth arrest in G, GA and GAP was not due to a starvation event resulting from the total consumption of the components of the G, GA or GAP media but to the exhaustion of essential nutrients originally present in the conidium.

Data presented above show that conidial swelling, rate of germination, germ tube morphology, septum formation and nuclear division were separate morphogenetic events differently influenced by the nutritional composition of the environment.

### 3.5. Transcriptome Analysis

**Figure 8:**
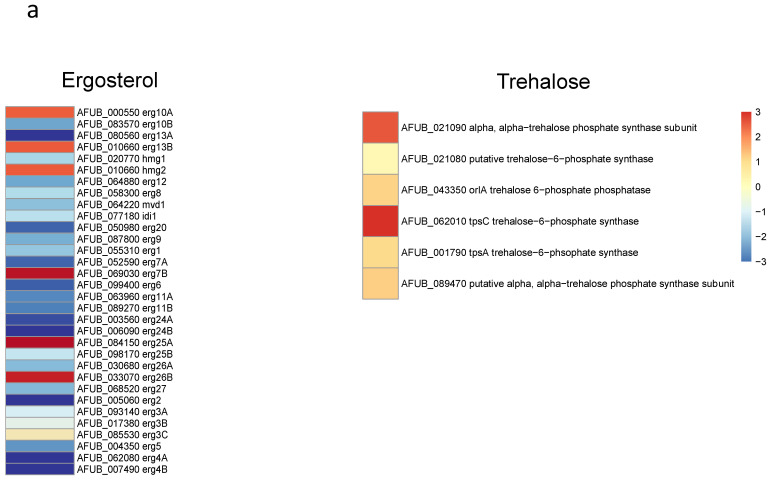

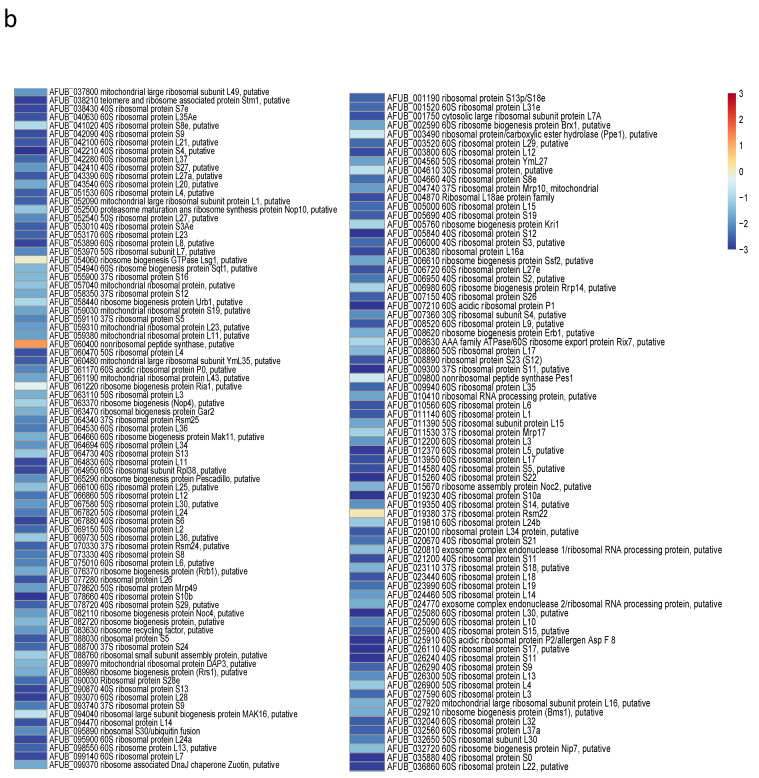

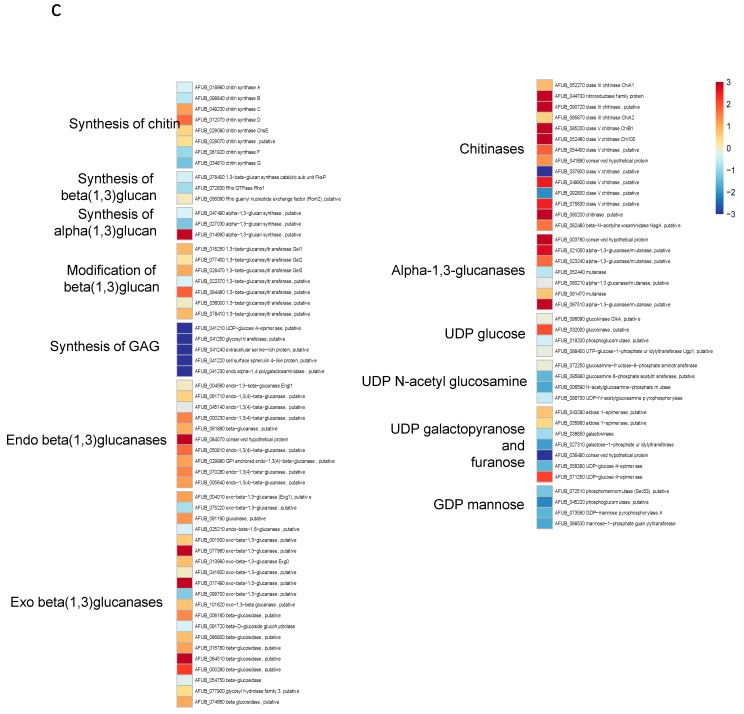

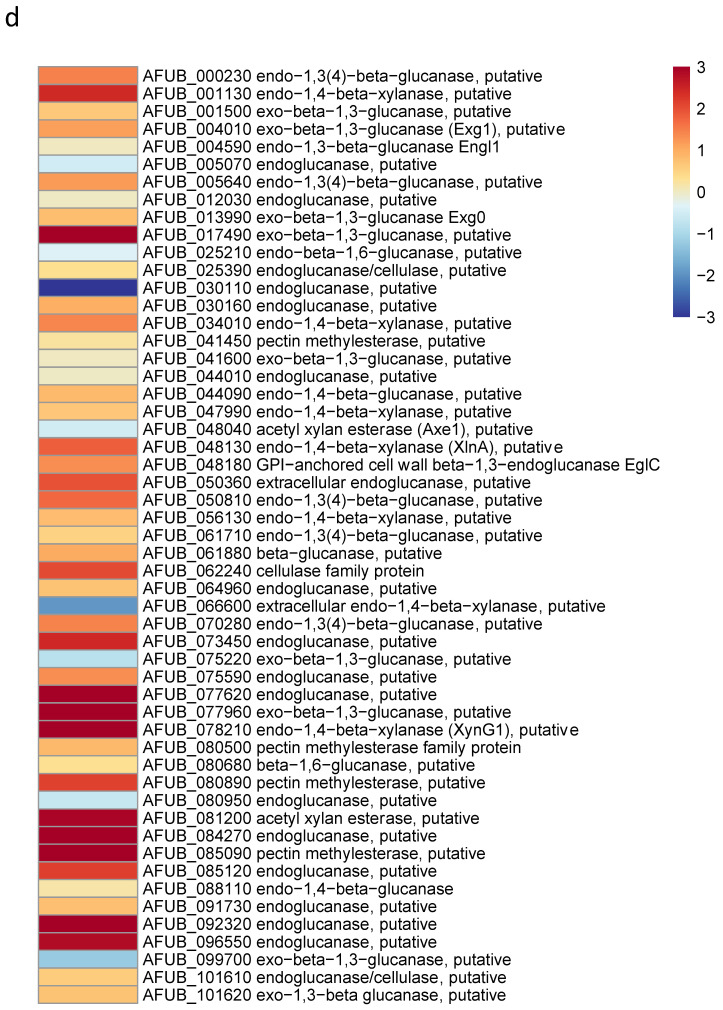
Heat maps showing significative examples of differentially regulated *A. fumigatus* genes during germination in GAP and MM media. Level of expression of genes for a ratio GAP/MM: (**a**) down regulation of ergosterol synthesis and upregulation of trehalose synthesis suggesting the entrance in quiescence of conidia germinating in GAP medium; (**b**) ribosome and protein synthesis pathways down regulated in absence of growth in GAP medium; (**c**) Down regulation of cell wall synthases and up regulation of autolytic cell wall glycosyl hydrolases indicating that the germ tubes are starved in GAP medium; (**d**) high expression of genes coding for plant material degrading glycosylhydrolases indicating the starvation status of GAP cells and the attemps to its quest for food.

Since the growth of germ tubes stopped after 30 h in GAP medium and the growth continued in MM medium, a transcriptome analysis was undertaken in these two media to attempt to identify which genes were essential for germ tube growth (Figure 8 and Supplementary Figure S8 and Table S1). A gene ontology analysis showed that, as expected, the growth arrest in GAP was directly associated with a reduced expression of the genes coding for ribosome and protein synthesis. Interestingly, the genes involved in the synthesis of B vitamin were all downregulated (Supplementary Table S1). As expected, the nucleotide sugar synthesis pathway and the alpha and beta glucan synthases were downregulated in the GAP medium. Deficit in growth was strongly associated with a downregulation of all pathways, leading to the production of GAG, which is a hallmark of *A. fumigatus* mycelial growth. In the GAP medium, nutrient starvation induced growth arrest and the fungal metabolism was tuned for nutritive anabolism. The search for food was correlated to the high expression of plant glycosyl hydrolases (such as cellulase, pectinase, hemicellulase) able to degrade plant material, indicating that the “grass eater” *A. fumigatus* was starved in this medium and actively looking for a carbon source. Interestingly, transcriptome data indicated that Zn was lacking during growth in GAP but not iron. The high expression of cell wall hydrolases such as alpha and beta glucanases as well as chitinases indicated that this fungus undertook autolysis to survive in this medium. The attempt of this fungus to survive in the GAP medium was also evidenced by the initiation of pathways to enter quiescence such as trehalose synthesis and a reduction in the membrane ergosterol synthesis. The entry into quiescence in the GAP medium was confirmed by the easy restart of growth when MM medium was added to the fungus incubated in GAP (supplementary Figure S7). The development of survival programming was associated with increased upregulation of many secondary metabolite clusters. With the exception of cluster 6, the genes controlling the formation of secondary metabolites from clusters 9, 12, 14, 16, 20, 31, 33 coding for unknown molecules but also clusters controlling the production of Endocrocin, Helvolic acid, Gliotoxin, Pyripyropene A and Neosartoricin Fumitremorgin were upregulated. These secondary metabolites are all putatively involved in the resistance of *A. fumigatus* to harmful co-inhabitants of the soil, such as protozoa, bacteria, insects and fungi [39,40,41,42,43].

### 3.6. Trying to Modify the Asynchrony of Conidial Germination in A.fumigatus

None of the nutritional conditions tested lead to modified synchronicity of germination. Moreover, all the mutants analyzed to date, including the 40 cell wall mutants of the *Aspergillus* unit and the over 400 transcription factor null mutants of the COFUN library [16], displayed an asynchronous germination. As an example, the germination parameters of the rodlet, melanin, α-(1,3)-glucan and rodlet/melanin/α-(1,3)-glucan mutants [14] were slightly modified by the respective deletions without abrogating asynchrony (Supplementary Table S2). Heterogeneity in germination was also seen when the germination experiments were undertaken with conidia of different ages or at different concentrations (Supplementary Figure S9 and Table S3). At the highest concentration (10^7^), half of the conidia remained quiescent whereas the other half were swollen conidia or germ tubes, suggesting that self-inhibitors were released by high concentrations of ungerminated conidia [44,45]. However, these self-inhibitors did not modify the asynchrony of conidial germination (Supplementary Figure S9).

**Figure 9:**
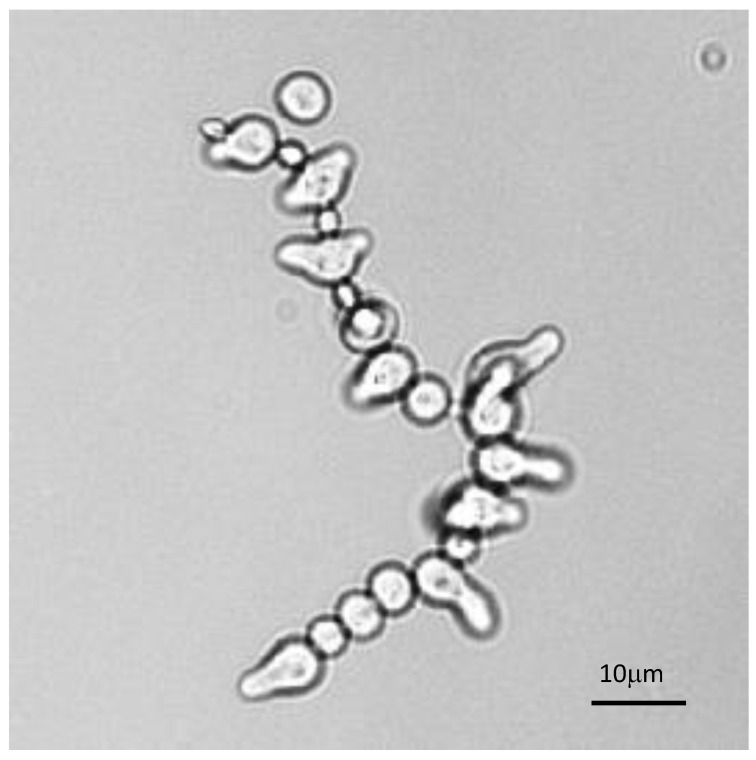
Asynchrony of conidial germination in a conidial chain of a glucanase mutant characterized by a defect in conidial separation. Note that the presence of resting, swollen or germinating conidia is totally independent of the conidium age.

The analysis of the germination of each conidium in conidial chains was also very informative. Such observation was facilitated in β1,3 glucanase mutants [46], which had defects in conidial separation. Therefore, during germination of these conidial chains, all conidia germinated differently, irrespectively of the age of the conidium (Figure 9). This result suggested that the asynchrony of germination was a characteristic inherent to the conidium which was established intracellularly prior to the release of the conidia into the atmosphere. Changing the environment did not abolish germination asynchrony, even though it can modify the values of the germination parameters.

**Figure 10:**
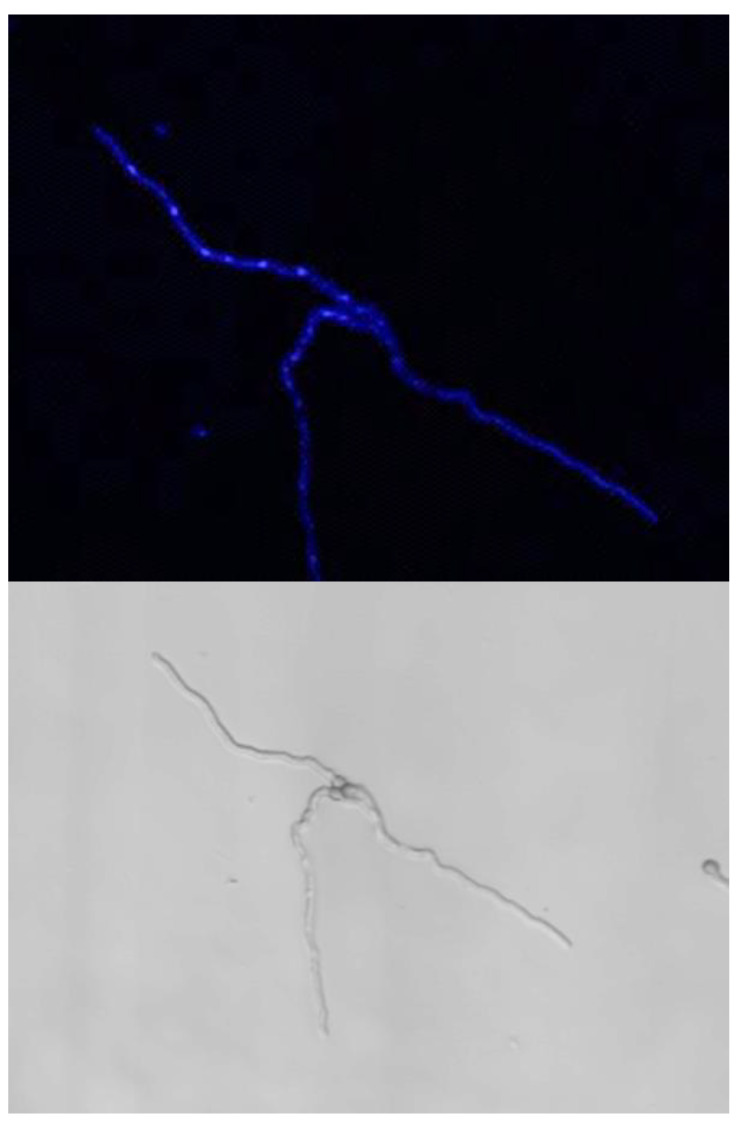
Stimulation of the germ tube growth by the GSK1324726A compound in the G or GA medium. The elongation of the germ tube was associated to an increase in nuclear divisions. Note the high number of nuclei per germinated conidia while in absence of drug the growth of the germ tube was stopped and contained only one nucleus per germ tube.

The use of various epigenetic inhibitors (RG108, Vorinostat, Romidespin, Zebularine, Trichostatin, Nanaomycin A, JQ1 Terbinafin, 3-deazaneplanocinA) added to G, GA, GAP and MM media did not modify the asynchrony of the germination (data not shown). Interestingly, one of these inhibitors, the tetrahydroquinoline GSK1324726A, stimulated conidial germination (30% and 76% germination in G or GA vs. G or GA + GSK1324726A, respectively, after 24 h), whereas the growth was similar in GAP and MM with or without GSK1324726A. The maximal size of the germ tube in G + GSK1324726A reached 98 µm after 24 h, whereas the maximal size was 25 µm in G after 24 h. Interestingly, in the G medium, the stimulation of growth by the GSK compound was associated with an increase in the nuclear division: 30% of germ tubes had five nuclei (Figure 10 and Supplementary Figure S10). However, none of them induced any modification of the germination heterogeneity, germ tube diameter or germ tube size/nucleus number correlation.

## 4. Discussion

Early transcriptome analyses have shown that active transcripts from one third of the genome are present in conidia. The analysis of these transcripts suggested that conidia were undergoing a fermentative metabolism at low noise [1,47]. These data suggest that resting conidia, resembling a time bomb, are ready to explode and germinate as soon as they encounter a favorable aqueous nutritive environment without any need for de novo transcription to initiate germination [2,48]. This has been confirmed by the present study, where germination was induced by the presence of a stimulatory environmental carbon and nitrogen source in absence of de novo RNA transcription. As there was no nuclear division in media containing glucose and (glucose + ammonium), the amount of RNA extracted was very limited and only reached in these two media 1% of the amount extracted when the fungus was grown in MM medium. The occurrence of low RNA levels in the G and GA media also confirmed that the transcripts present in the resting conidia were sufficient without de novo transcription to produce enough proteins to initiate germination after glucose ± ammonium was sensed [1]. In contrast, nuclear division was active in the germinating conidia of both GAP and MM media. RNA-seq analysis of the transcripts regulated during germ tube emergence showed that the genes upregulated during germination were highly similar to those upregulated during vegetative growth in other studies [47,48,49]. Interestingly, the blockade of growth during germination was associated with the stimulation of the expression of genes induced by starvation, such as the glycoside hydrolases responsible for the hydrolysis of plant polysaccharides, which represent a natural substrate for this fungus, living in the soil on decaying plant material. The follow-up of the expression of secondary metabolite genes also suggested that these metabolites may have a yet undiscovered role in the fungus search of energy. Sensing of the exogenous nutrients by the conidium was enough to trigger germination, while the follow-up of polarized germ tube growth was fully dependent on the presence of appropriate exogenous nutrients. The mechanisms implemented by a conidium to sense the environment to initiate germination remain, however, unknown.

Our study confirmed that nuclear division and cytokinesis are not closely coupled during conidial germination. This is also true in apicomplexan parasites, where replication in these organisms relies on the mechanisms in place to correctly segregate complete sets of organelles [50]. Tip growth in hyphae is a continuous process of extension which is not coupled to nuclear division in the same manner that bud formation and mitosis are connected in yeast. When *Neurospora crassa* and *Podospora anserina*, *A. nidulans* or *Ashbya gossypii* hypha extend, the population of nuclei increases through asynchronous or parasynchronous mitosis, and the formation of new cells, or hyphal compartments, does not seem tightly coordinated to nuclear division [51,52,53,54].

Experimental results in mold research are commonly represented as average measurements across whole populations of conidia whilst ignoring what is happening at the single cell or subpopulation level within these populations [55]. The present study has, however, shown the heterogeneity of germination patterns of individual conidia in a conidial population. Bleichrold et al. [9] already showed that the conidial cell wall is heterogeneous in composition among different conidia originating from a single conidium and that this heterogeneity is modified by germination. Heterogeneity can be a result of the cell cycle, cell ageing, epigenetic regulation [56,57], cellular metabolism [58] and gene expression stochasticity [59]. This stochasticity itself can also be regulated [60]. In *A. fumigatus*, the fact that CFW labeling levels of individual conidia were not inherited in their offspring suggested that many genes that are directly involved in cell wall synthesis are differentially regulated, resulting in cell wall heterogeneity [9]. The fact that the progeny of a colony issued from a quick or slow germinating conidium displayed the same germinating asynchrony as their parental conidia suggests that this asynchrony is not fixed genetically since all nuclei that originate from single mitotic events should have identical DNA packages.

The concept of nuclear autonomy is now accepted for multinucleate organisms [51,61,62,63] but has not been properly investigated in *A. fumigatus.* The putative role of epigenetics on germination asynchrony should be investigated, especially since the genome of *A. fumigatus* has been shown to be rich in epigenetic markers and in DNA modification enzymes (http://www.fungidb.org) [64]. Epigenetic processes have been shown to be involved in heterogeneous expression of hyphae [65]; however, our study with inhibitors does not suggest a role for epigenetics in germination asynchrony. The impact of the GSK1324726A molecule on conidial germination was even unexpected. This molecule belongs to a family of inhibitors of Bromodomain and extraterminal (BET) family proteins known to interfere with transcriptional initiation after binding to lysine acetylated histones and elongation, used to inhibit the growth of cancer cells. Moreover, the fungal BET proteins in yeast are global transcriptional regulators which regulate the transcription of >500 genes and which are essential for fungal life. However, JQ1, which is another member of the same inhibitor family, has no effect on *A. fumigatus*, and the human BET inhibitors do not seem to have an effect in yeast [66,67]. The reasons for the stimulatory effect (germination and germ tube length) of the GSK compound on *A. fumigatus* in nutrient deficient media (G and GA) remain unknown.

In *A. gossypii*, it has been shown that nuclei display independence in a common cytoplasm [53]. The mechanism to produce spatially variable transcripts and heterogeneous cell behaviors is at least partly associated with the segregation of non-translating mRNA molecules and their associated proteins into messenger ribonucleoprotein (mRNP) granules [68,69,70]. The best characterized of these mRNPs are processing bodies (P-bodies), which are liquid droplet-like compartments that lack a limiting membrane and contain specific sets of proteins and mRNAs [71,72,73]. It has been proposed that P-bodies contain translationally repressed mRNAs in combination with signaling molecules important for the proper control of cell proliferation, environmental response and morphological switching. A putative role of these mRNPs in the asynchrony in germination is currently being investigated.

Why would a multinucleated cell evolve mechanisms to ensure asynchronous nuclear division? Phenotypic heterogeneity in conidial survival has been observed during environmental stress [74,75,76,77]. Interestingly, a delay in germination has been associated with drug tolerance [9,16]. Slow-growing cells are better in tolerating adverse environments. This is consistent with non- or slow-growing bacteria that tolerate both complement and antibiotics [78,79,80]. A nuclear autonomous cycle also enables nuclei in a specific subcellular location to divide, such as near a branch point or at a site rich in nutrients, without having to duplicate or transport more distant nuclei. It may also allow differential resistance and be responsible for the source of latent infections. The role of conidial asynchrony during infection has not been explored yet.

## Figures and Tables

**Table 1:** Germination parameters in different media G, GA, GAP and MM.

	G	GA	GAP	MM
Conidia area at T0, mean, px	263 ± 9 ^a^	272 ± 13 ^a^	253 ± 21 ^a^	254 ± 17 ^a^
Conidia area at 6 h, px	355 ± 10 ^b^	378 ± 13 ^b^	416 ± 21 ^a^	519 ± 17 ^a^
Conidia area at 8 h, px	395 ± 10 ^c^	412 ± 13 ^c^	518 ± 13 ^b^	757 ± 21 ^a^
Conidia area at 16 h, px	471 ± 10 ^c^	460 ± 16 ^b^	1078 ± 24 ^a^	NA
Conidia area at first germ tubes appearance, px	620 ± 52 ^c^	545 ± 29 ^c^	1435 ± 39 ^a^	1184 ± 16 ^b^
Time at first germ tube appearance, min	1223 ± 51 ^a^	834 ± 28 ^b^	843 ± 38 ^b^	528 ± 15 ^c^

Measurements are recorded in pixels (px). Mean ± standard error are presented. Levels not connected by same letter are significantly different. NA: not appropriate because, in the MM medium, 93% of conidia have germinated and produced mycelium by this time.

## Data Availability

The data presented in this study are available on request from the corresponding author.

## Supplementary Materials

The following are available online at https://www.mdpi.com/2309-608X/7/1/30/s1, Figure S1: Absence of correlation between the isotropic swelling rate and the time at the beginning of the swelling (a) or at the emission of the germ tube (b) on twenty conidia in minimal medium; Figures S2–S5: Videos showing the conidial germination in different media (see below); Figure S6: Characteristics of the conidial germination of *A. fumigatus* conidia in the different media glucose (G), glucose-ammonium (GA), glucose-ammonium-phosphate (GAP), minimal medium (MM) and RPMI; Figure S7: Germinating conidia were alive after they stopped growth in a 1% Glucose medium; Figure S8: Heat map of expression of genes coding for secondary metabolite clusters differentially regulated during germination in GAP and MM media; Figure S9: Increase in conidial concentration reduces the germination; Figure S10: Stimulation of the germination by the GSK1324726A drug. Table S1: transcriptome analysis of differentially regulated *A. fumigatus* genes during germination in GAP and MM media; Table S2: Germination parameters of the conidia of parental strains (KU80) and alpha 1,3 glucan mutant (A), Melanin mutant (P) rodlet mutant (R) and quintuple mutant without melanin, rodlets and alpha 1,3 glucan (APR); Table S3: Germination of conidia recovered after 1 (W1), 2 (W2), 4 (W4) and 8 (W8) weeks of growth. Video S1: Germination of conidia in G medium, Video S2: Germination of conidia in GA medium, Video S3: Germination of conidia in GAP medium, Video S4: Germination of conidia in MM medium.
