# Supplementary material for: Aspergillus fumigatus, One Uninucleate Species with Disparate Offspring
Source: J Fungi (Basel). 2021 Jan 6;7(1):30. doi: 10.3390/jof7010030 (PMC7825634; doi:10.3390/jof7010030)
Supplement: Supplementary file 1 [file jof-07-00030-s001.zip › Supplementary Figures_danion_germination_120208revised FD22122020.pptx]

## Slide 1
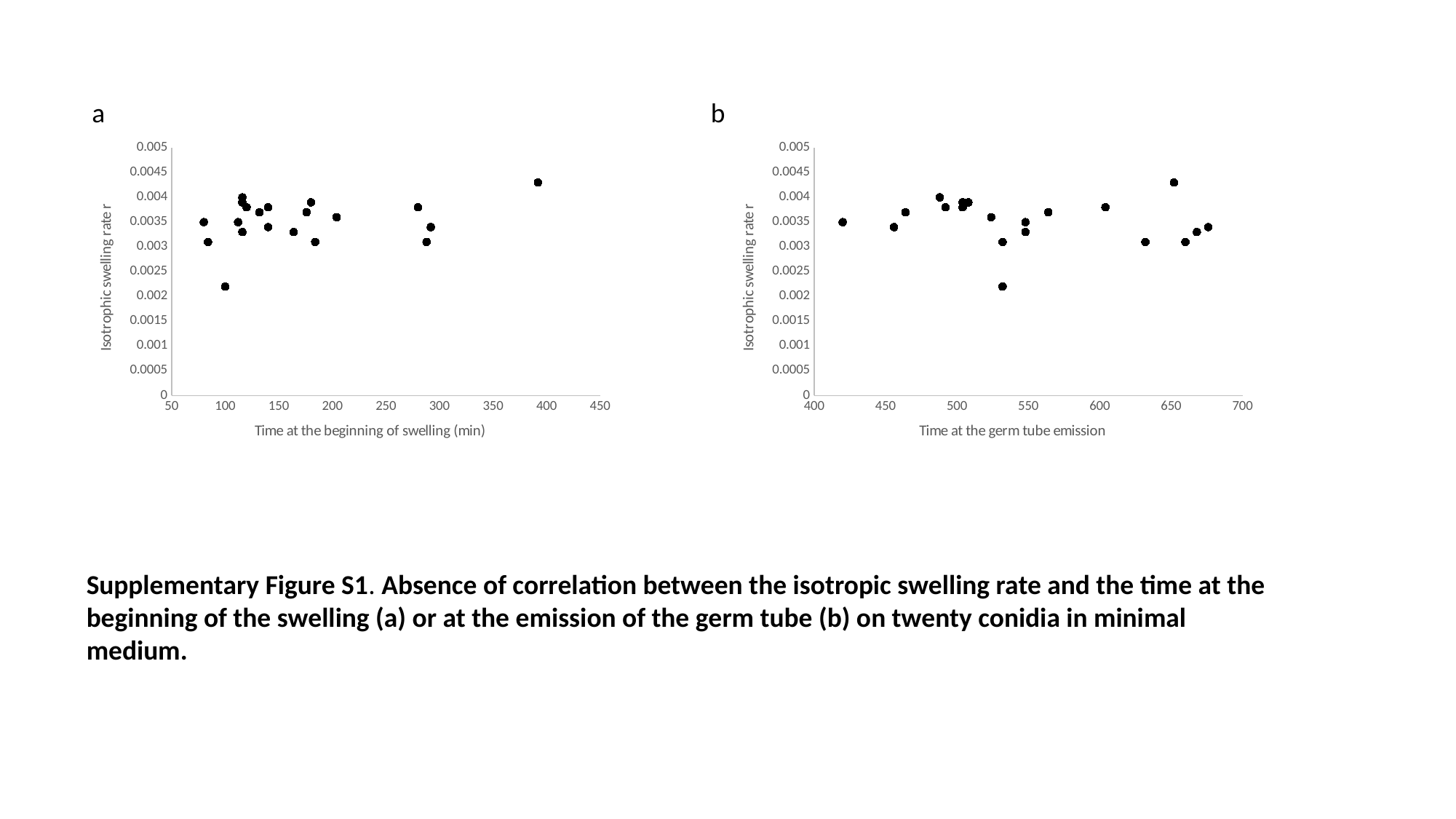

a
b
### Chart
| Category | |
|---|---|
### Chart
| Category | |
|---|---|Supplementary Figure S1. Absence of correlation between the isotropic swelling rate and the time at the beginning of the swelling (a) or at the emission of the germ tube (b) on twenty conidia in minimal medium.

## Slide 2
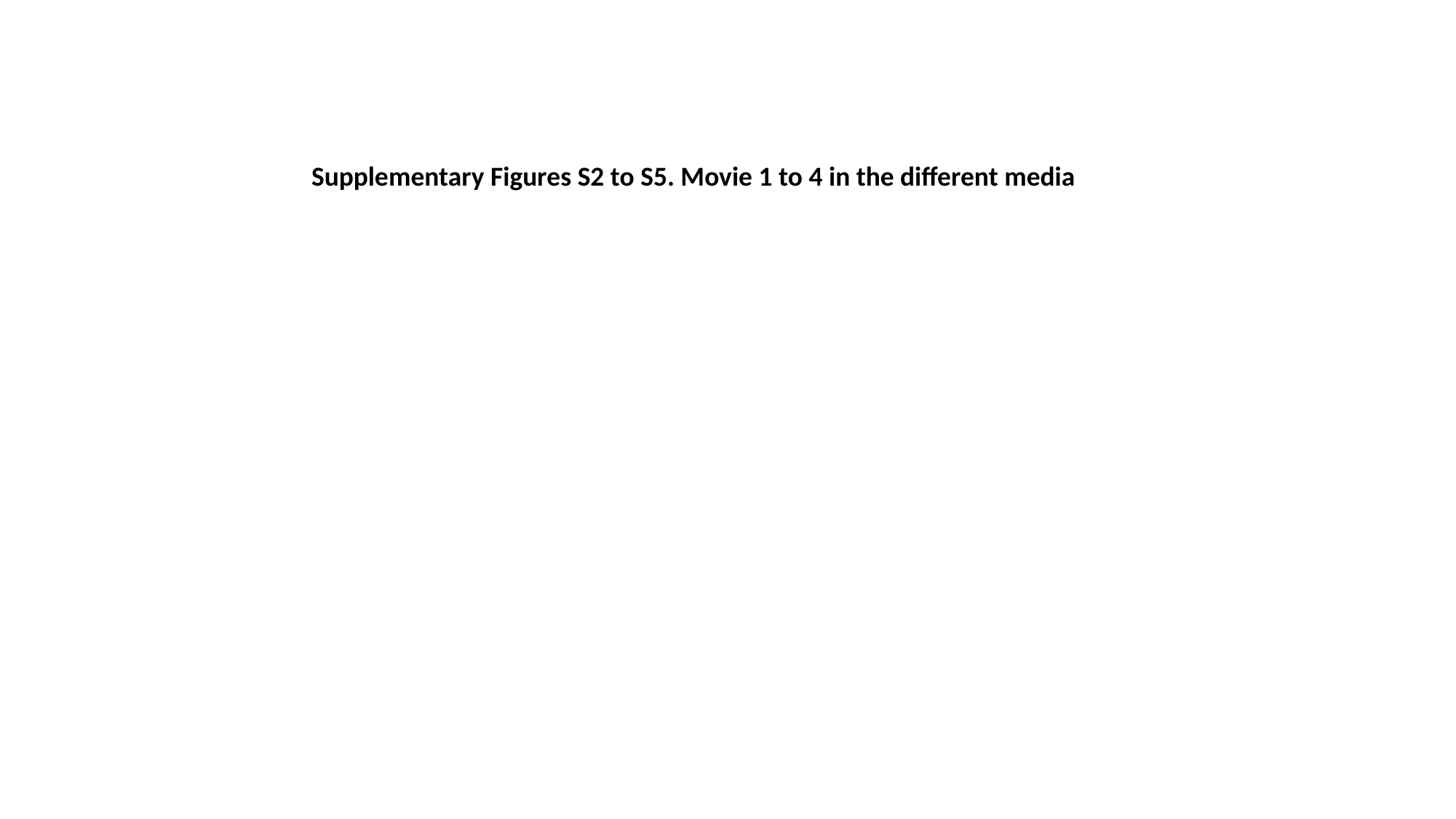

Supplementary Figures S2 to S5. Movie 1 to 4 in the different media

## Slide 3
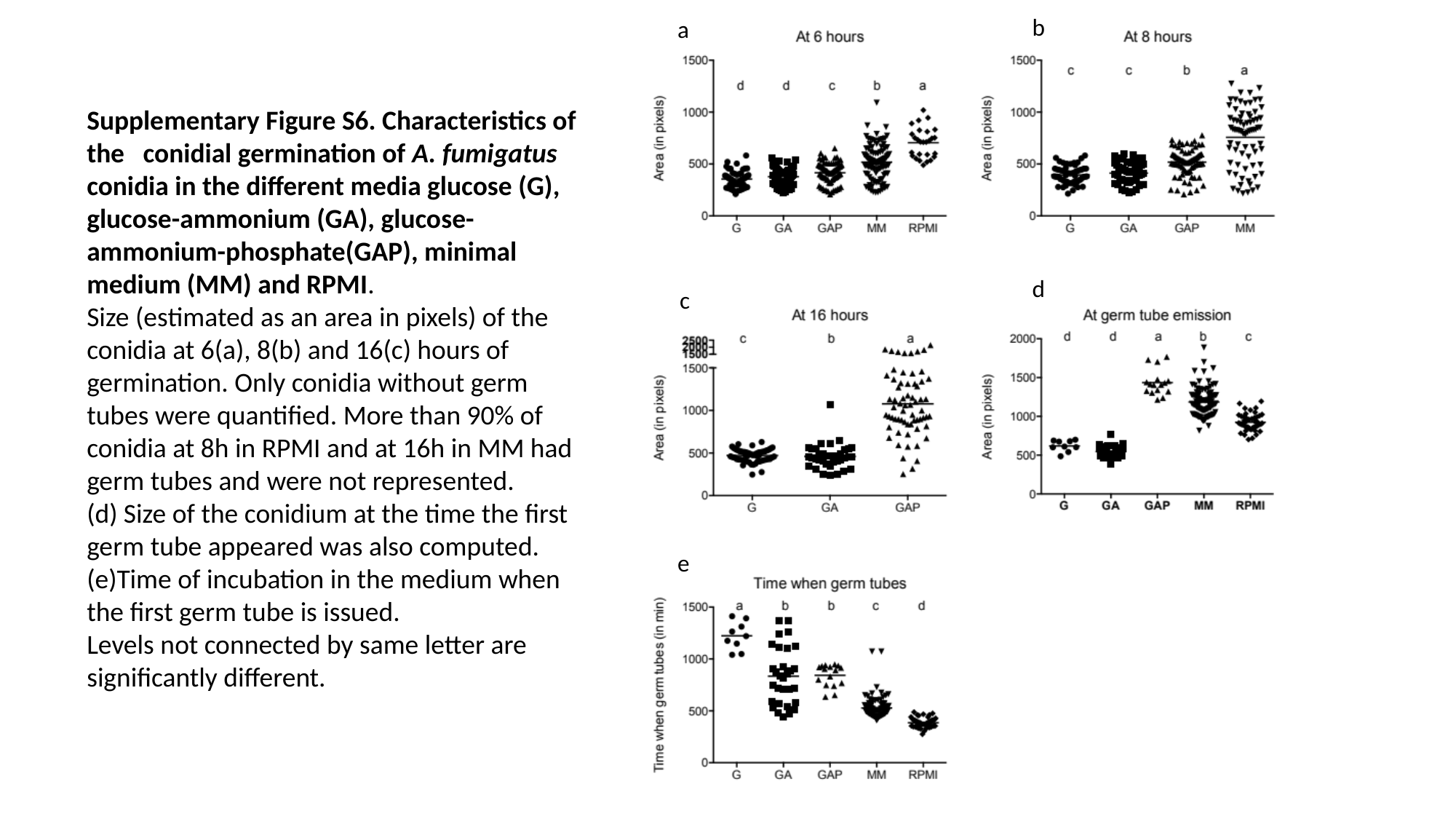

b
a
Supplementary Figure S6. Characteristics of the conidial germination of A. fumigatus conidia in the different media glucose (G), glucose-ammonium (GA), glucose-ammonium-phosphate(GAP), minimal medium (MM) and RPMI.
Size (estimated as an area in pixels) of the conidia at 6(a), 8(b) and 16(c) hours of germination. Only conidia without germ tubes were quantified. More than 90% of conidia at 8h in RPMI and at 16h in MM had germ tubes and were not represented.
(d) Size of the conidium at the time the first germ tube appeared was also computed. (e)Time of incubation in the medium when the first germ tube is issued.
Levels not connected by same letter are significantly different.
d
c
e

## Slide 4
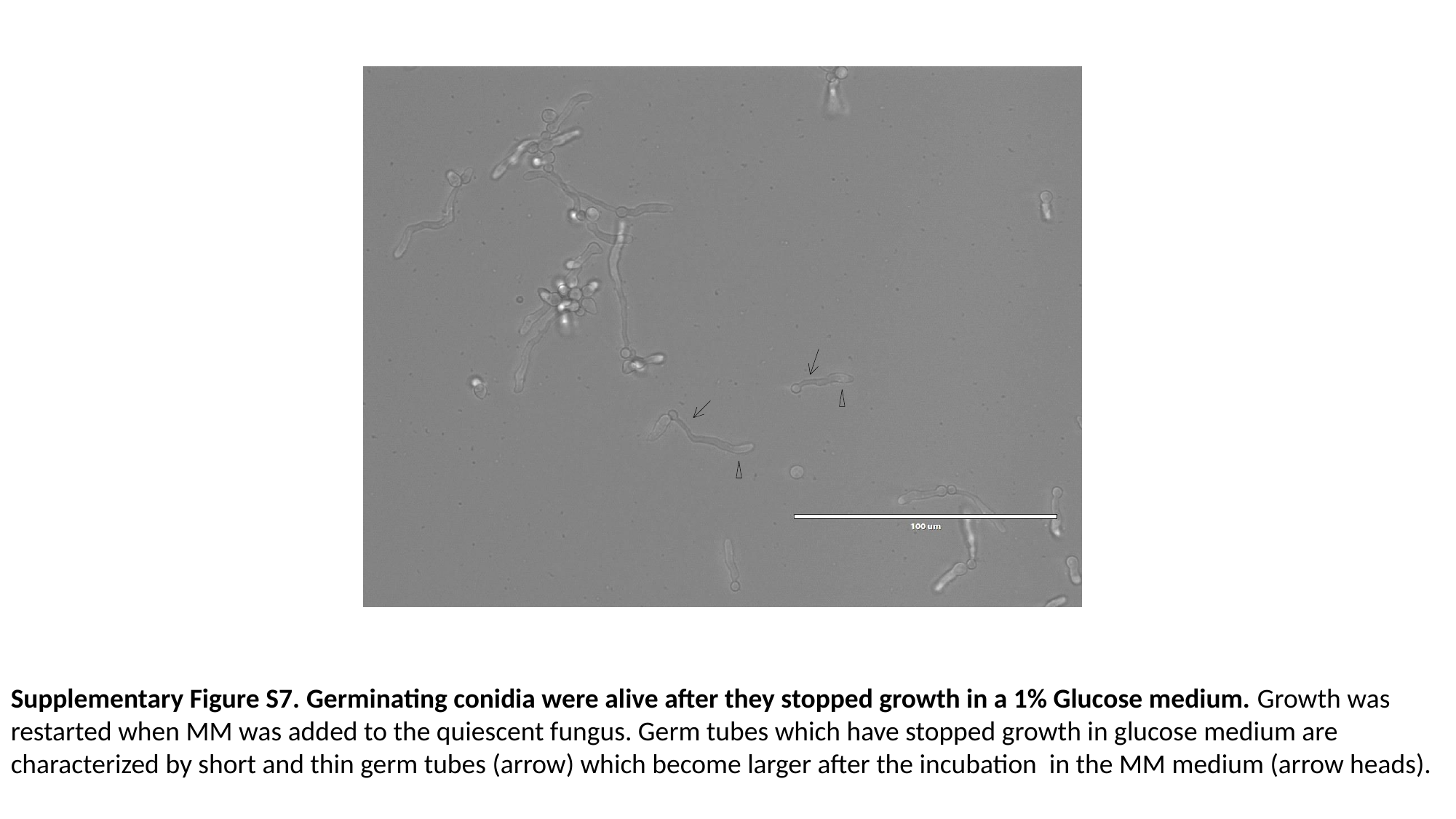

Supplementary Figure S7. Germinating conidia were alive after they stopped growth in a 1% Glucose medium. Growth was restarted when MM was added to the quiescent fungus. Germ tubes which have stopped growth in glucose medium are characterized by short and thin germ tubes (arrow) which become larger after the incubation in the MM medium (arrow heads).

## Slide 5
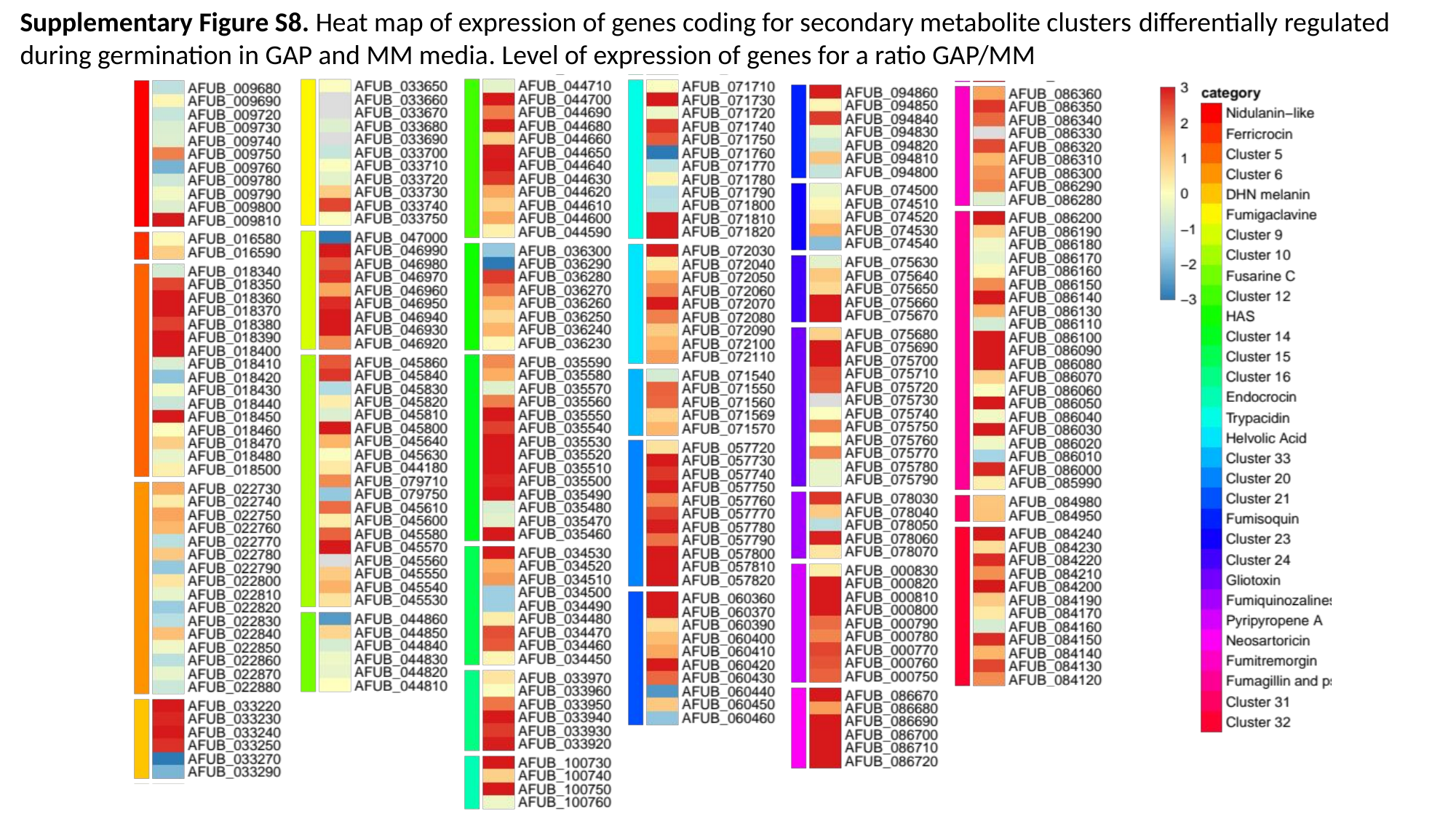

Supplementary Figure S8. Heat map of expression of genes coding for secondary metabolite clusters differentially regulated during germination in GAP and MM media. Level of expression of genes for a ratio GAP/MM

## Slide 6
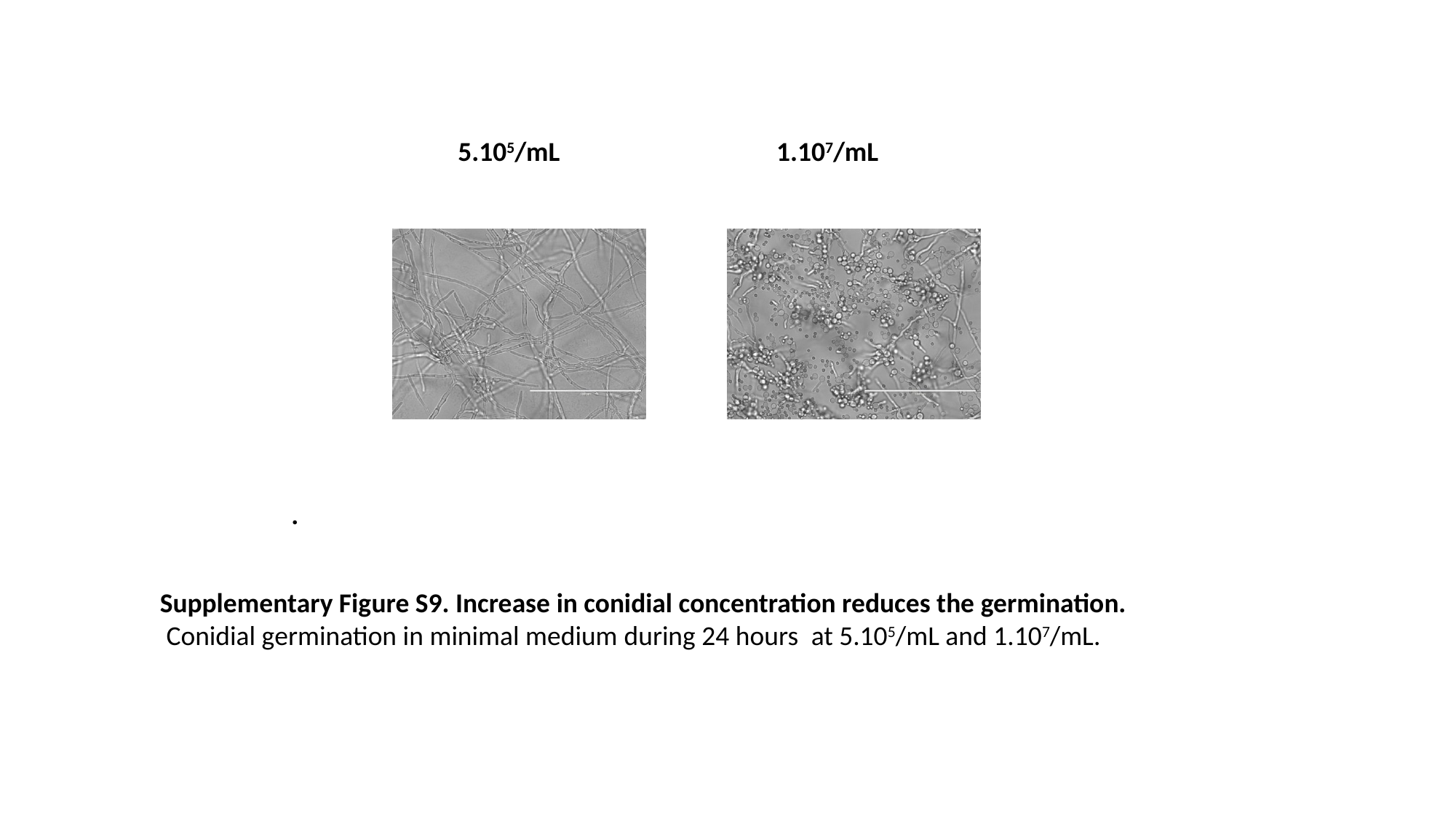

5.105/mL
1.107/mL
.
Supplementary Figure S9. Increase in conidial concentration reduces the germination.
 Conidial germination in minimal medium during 24 hours at 5.105/mL and 1.107/mL.

## Slide 7
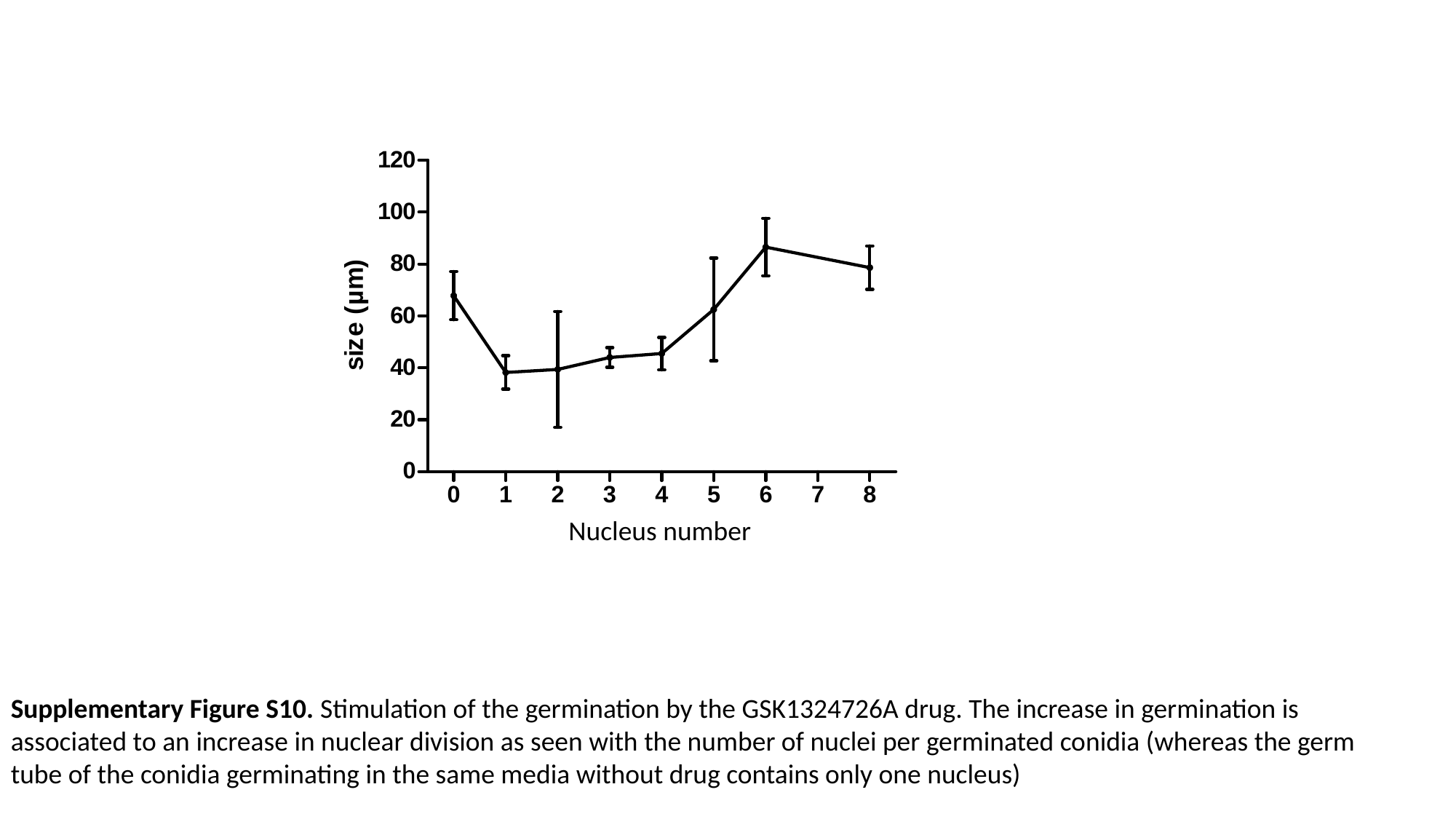

Nucleus number
Supplementary Figure S10. Stimulation of the germination by the GSK1324726A drug. The increase in germination is associated to an increase in nuclear division as seen with the number of nuclei per germinated conidia (whereas the germ tube of the conidia germinating in the same media without drug contains only one nucleus)
