# Supplementary material for: Aspergillus fumigatus, One Uninucleate Species with Disparate Offspring
Source: J Fungi (Basel). 2021 Jan 6;7(1):30. doi: 10.3390/jof7010030 (PMC7825634; doi:10.3390/jof7010030)
Supplement: Supplementary file 1 [file jof-07-00030-s001.zip › Supplementary Tables S2 and S3 ms Danion26 8vs21122020.docx]

Supplementary Table S2 Germination parameters of the conidia of parental strains (KU80) and alpha 1,3 glucan mutant (A), Melanin mutant (P) rodlet mutant ® and quintuple mutant without melanin, rodlets and alpha 1,3 glucan (APR).

|  | KU80 | A | P | R | APR |
| --- | --- | --- | --- | --- | --- |
| Conidia area at T0, mean, px | 270 ±8^a^ | 285 ±7^a^ | 272 ±7^a^ | 247 ±10^a^ | 271 ±9^a^ |
| Conidia area at 6h, px | 510 ±38^a^ | 617 ±30^c^ | 546 ±26^a^ | 529 ±28^b^ | 716 ±32^d^ |
| Conidia area when germ tubes, px | 1156 ±33^a^ | 1056 ±26^b^ | 1204 ±23^a^ | 1120 ±27^b^ | 981 ±26^c^ |
| Time at germ tube, (min) | 576 ±13^a^ | 480 ±10^c^ | 519 ±9^b^ | 489 ±10^c^ | 398 ±10^d^ |

Mean ±standard error are presented and levels not connected by same letter are significantly different

Supplementary Table S3. Germination of conidia recovered after 1 (W1), 2 (W2), 4 (W4) and 8 (W8) of growth.

|  | W1 | W2 | W4 | W8 |
| --- | --- | --- | --- | --- |
| Conidia area at T0, mean, px | 265 ±36^a^ | 256 ±42^a^ | 260 ±30^a^ | 266 ±33^a^ |
| Conidia area at 6h, px | 537 ±36^a^ | 569 ±40^a^ | 499 ±30^a^ | 462 ±33^b^ |
| Conidia area at 8h, px | 851 ±39^a^ | 927 ±42^a^ | 820 ±30^a^ | 700 ±33^b^ |
| Conidia area when germ tubes, px | 1332 ±37^a^ | 1416 ±40 ^a^ | 1210 ±30^b^ | 1160 ±33^b^ |
| Time at germ tube, min | 545 ±15^b^ | 554 ±16^b^ | 555 ±12^b^ | 588 ±13^a^ |

Mean ±standard error are presented and levels not connected by same letter are significantly different
